# Residual unabsorbed midazolam: a case report

**DOI:** 10.1186/s13256-023-03817-0

**Published:** 2023-03-25

**Authors:** Hemanth A. Baboolal, A. Gulati

**Affiliations:** 1 Department of Anesthesiology, University of North Carolina Hospital, 101 Manning Drive, Chapel Hill, NC 27514 USA; 2 Department of Pediatrics, University of North Carolina, Chapel Hill, USA

**Keywords:** Midazolam: pediatrics oral dosage, Premedication: side effects, Benzodiazepines: respiratory effects, Gastric volume: PO liquid effect

## Abstract

**Background:**

Oral midazolam is commonly administered to reduce anxiety in children presenting for medical procedures or surgery. It is unclear what volume of medication remains unabsorbed in the stomach when the child presents for anesthetic induction prior to these procedures. The presence of any significant residual medication in the stomach has significant clinical implications in the postoperative period.

**Case presentation:**

A 5-year-old white Caucasian boy presented for upper gastrointestinal endoscopy after receiving oral midazolam liquid. Insertion of the endoscope into the stomach revealed a significant amount of unabsorbed medication remaining within the gastric cavity.

**Conclusion:**

Clinicians should be aware that the sedative effects of midazolam may be present before the medication is fully absorbed. A significant amount of unabsorbed medication may be present in the stomach during medical procedures/surgery. This may continue to be absorbed in the intraoperative and postoperative period, with unwanted clinical effect.

## Introduction

Oral midazolam is frequently administered as premedication to anxious pediatric patients prior to surgical or invasive procedures. However this medication has several disadvantages in the postprocedure period such as excessive drowsiness, emergence delirium and agitation. Medication that remains in the stomach after anesthetic induction continues to be absorbed into the bloodstream and increases the likelihood of unwanted adverse effects. We present the case of a child who received oral midazolam and accompanying images demonstrating a significant volume of unabsorbed midazolam in the stomach.

## Case

**Fig. 1:**
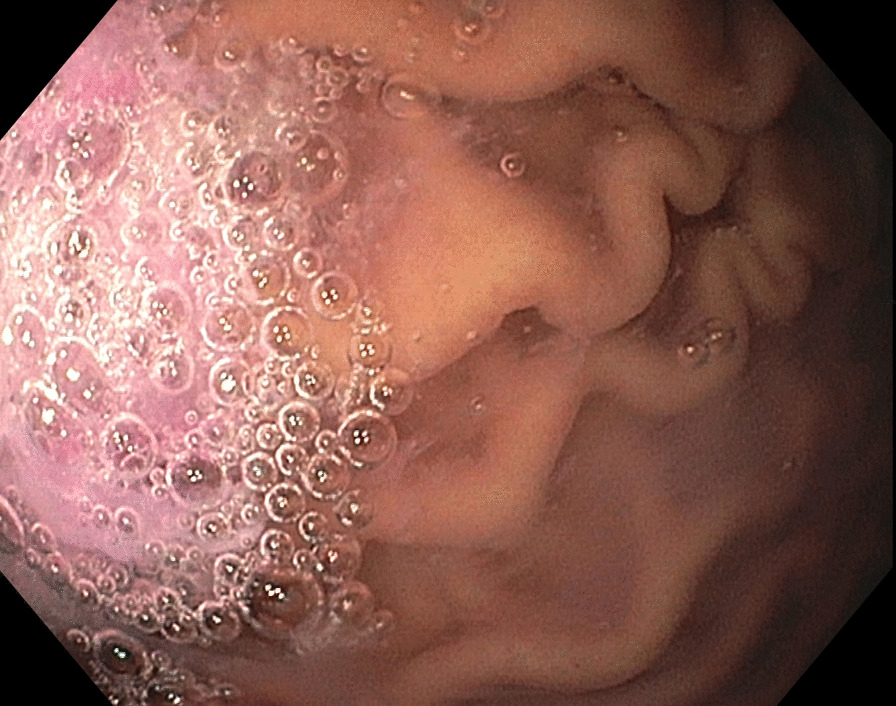
Image of stomach demonstrating pink residual unabsorbed medication

**Fig. 2:**
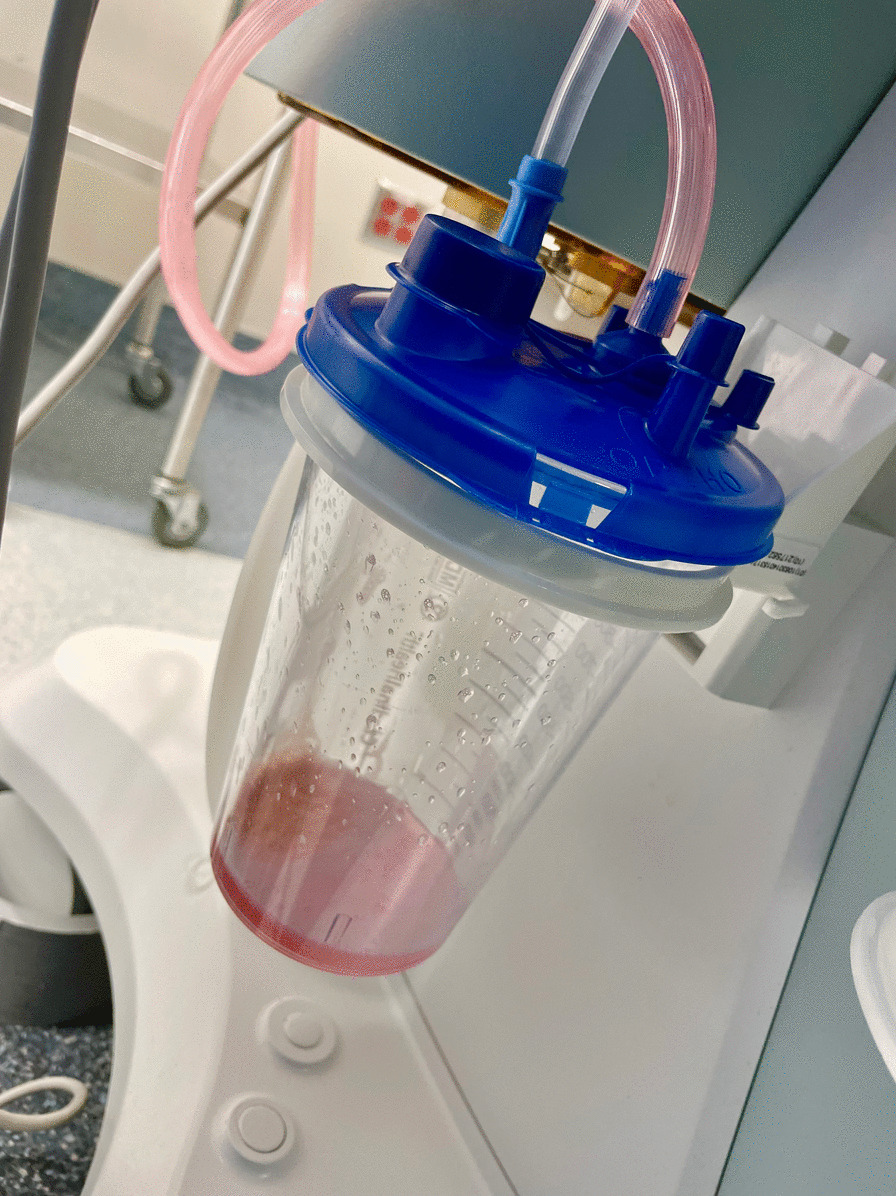
Pink fluid suctioned from patient’s stomach

A 5-year-old 19 kg white Caucasian boy without significant medical history presented for gastrointestinal endoscopy to investigate vague upper abdominal pain. The pain was diffuse, mild in severity, had a deep aching nature, and was not related to meals. It occurred every 2–3 days, lasted for a few minutes at a time, and usually resolved spontaneously. He did not take any regular medication and had no previous surgery or interventions. There was no family or psychosocial history of note. Physical examination was normal. Specifically, there was no abdominal tenderness on exam, no masses were palpated, and bowel sounds were normal. The child had not had anything to eat or drink for 10 hours prior to the procedure. He appeared anxious and was therefore given 10 mg oral midazolam (0.52 mg/kg, total volume 5 ml), with the usual dose of oral midazolam in children being 0.5 mg/kg. No carrier agent such as water or juice was given with the medication. Once he became calm approximately 45 minutes later, he was taken to the procedure room to induce anesthesia. Although the standard in some countries is to allow 1–2 hours to pass after drinking fluids prior to surgical procedures, it is the norm at our institution to make an exception for ingested medication. He was notably drowsy but rousable to speech, he was able to answer some questions correctly but was not fully oriented. This corresponded to a Ramsay sedation score of 2. He was calm and cooperative during a sevoflurane mask induction. After induction, he received a Propofol infusion to maintain anesthesia. No other medications were administered. The endoscope was inserted into the stomach and the image below was obtained (Fig. 1). The image demonstrates a significant volume of midazolam (pink in color) remaining in the stomach. The suctioned liquid (Fig. 2) was compared with the oral midazolam mixture and was visually identical. The procedure was completed after approximately 30 minutes and the child was transported to the recovery room. On arrival to the recovery room, the child was still sedated. He responded to stimulation by jaw thrust, but did not respond to voice or light touch (Ramsay sedation score of 5). He underwent standard cardiorespiratory monitoring, in addition to monitoring depth of sedation. He roused spontaneously approximately 40 minutes after arrival, at which point he was calm, awake, answering questions, and oriented. Subsequent gastrointestinal investigations and a gastric emptying study were normal. An abdominal ultrasound exam was also normal. The pain resolved approximately 2 months later and did not recur on follow-up visits.

## Discussion

Oral midazolam is frequently administered to children prior to surgery to facilitate separation from parents and ensure a calm anesthetic induction [1, 4]. However, midazolam premedication has well-documented potential adverse effects, including respiratory depression, airway obstruction, prolonged emergence, emergence agitation, and delayed discharge from the recovery room [1]. The time to maximum serum concentration after oral administration is 1–1.5 hours, with an elimination half-life ranging to almost 4 hours [2]. Oral bioavailability varies widely from 21% to 66%, therefore it should not be surprising to find significant unabsorbed medication in the stomach [3]. However, what is surprising in this case is the child’s significant level of sedation, although it appeared on endoscopy that only a small amount had been absorbed.

When children are brought to the operating room after receiving oral midazolam, there is no easily performed study that would demonstrate what proportion of medication has been absorbed, versus the amount that remains in the stomach. The clinician can reasonably expect that residual unabsorbed midazolam continues to be absorbed into the bloodstream, in keeping with traditionally accepted pharmacokinetics of oral medication [3].

We present this case to demonstrate that a child receiving oral premedication with midazolam may have an unexpectedly large volume of unabsorbed medication in the stomach, despite having the desired clinical effects. We propose, on the basis of our findings, that the physician should not assume that the medication is fully absorbed, although the child may appear sedated. This child was calm and well-sedated when he was brought to the procedure room. It is difficult to know if he would have become more sedated with time, or if drug effect had plateaued at the time of anesthesia induction. The image demonstrates that desired clinical effect may be present despite absorbing only a small amount of administered midazolam. Once a child has had an anesthetic induction, continued absorption of midazolam provides no desired clinical benefit during the procedure or in the postoperative period. It also increases the likelihood that the child will experience adverse effects in the recovery room [4].

The fluid suctioned from the stomach was not sent to a laboratory for chemical analysis primarily because this was of academic interest, would have incurred additional expense for the patient, and would not have changed management. It is possible that the pink liquid may have contained preservative and a lesser concentration of midazolam than the administered dose.

We emphasize that unabsorbed medication will very likely continue to be absorbed during the intraoperative and postoperative period. Awareness of this issue is important. It should be considered, especially in the case of a child who has unexpectedly prolonged emergence from anesthesia, or the child that remains drowsy for an extended period of time in the postoperative recovery room. This has significance in clinical situations where midazolam has been administered by non-anesthesiologists who may not be aware of this issue.

Further clinical studies are necessary before presuming that this occurs in all pediatric patients. Research is also necessary to decide if there are potential advantages in suctioning remaining medication out of the stomach. The risks of placing an orogastric tube include trauma to upper airway structures, bleeding, and incorrect placement. The risks and benefits should therefore be weighed and considered on a case by case basis. If the child is already undergoing a procedure where the gastric cavity is visualized as part of the procedure, then it would be reasonable to remove this medication from the stomach.

## Conclusion

Clinicians should be aware that the sedative effects of liquid midazolam may be present before the medication is fully absorbed. A significant amount of unabsorbed medication may be present in the stomach during medical procedures or surgery. This has important clinical significance because residual gastric medication may continue to be absorbed in the intraoperative and postoperative period, with unwanted clinical effects.

## Data Availability

Not applicable.
